# Application of Industrial Waste Materials by Alkaline Activation for Use as Geopolymer Binders

**DOI:** 10.3390/ma16247651

**Published:** 2023-12-14

**Authors:** Kinga Setlak, Janusz Mikuła, Michał Łach

**Affiliations:** Faculty of Materials Engineering and Physics, Cracow University of Technology, Jana Pawła II 37, 31-864 Cracow, Poland; janusz.mikula@pk.edu.pl (J.M.); michal.lach@pk.edu.pl (M.Ł.)

**Keywords:** waste materials, alkali-activated binders, chalcedonite, diatomite, amphibolite, fly ash from Bełchatów, metakaolin

## Abstract

The purpose of this study is to synthesize geopolymer binders as an environmentally friendly alternative to conventional cement using available local raw materials. Waste materials such as chalcedonite (Ch), amphibolite (A), fly ash from lignite combustion (PB), and diatomite dust (D) calcined at 900 °C were used to produce geopolymer binders. Metakaolin (M) was used as an additional modifier for binders based on waste materials. The base materials were subjected to fluorescence X-ray fluorescence (XRF) analysis and X-ray diffractometry (XRD) to determine chemical and phase composition. A laser particle size analysis was also performed. The various mixtures of raw materials were activated with a 10 M solution of NaOH and sodium water glass and then annealed for 24 h at 60 °C. The produced geopolymer binders were conditioned for 28 days under laboratory conditions and then subjected to microstructural analysis (SEM) and flexural and compressive strength tests. The best compressive strength results were obtained by the Ch + PB samples—more than 57 MPa, while the lowest results were obtained by the Ch + D+A + M samples—more than 20 MPa. On the other hand, as a result of the flexural strength tests, the highest flexural results were obtained by D + A + M + PB binders—more than 12 MPa, and the lowest values were obtained by binders based on Ch + D+A + M—about 4.8 MPa.

## 1. Introduction

The primary binder used in the construction sector is cement. The Portland cement production process requires very high energy and raw material use, emitting significant amounts of CO_2_ [1]. According to a report by Statista, global cement production in 2030 could reach as much as 4.83 billion metric tonnes. Thus, it is estimated that there will be an increase of more than 10% in global cement production in that period compared to 2020 [2]. This has caused an increasingly intensive search for alternatives to Portland cement. The emergence of one possibility in the form of geopolymer materials will make it possible to reduce CO_2_ emissions by using waste materials from industry (fly ash or blast furnace slag) and turning them into a binder material [3,4,5]. Waste research is mainly driven by the need to conserve scarce natural resources, reduce environmental pollution, and save energy [6,7]. Geopolymer materials are obtained by polycondensation of aluminum silicates and are characterized by an amorphous or semi-crystalline structure. The tetrahedral combination of [SiO_4_]^4−^ and [AlO_4_]^5−^-based aluminosilicates forms the spatial structure of the geopolymer network, which is linked to each other by oxygen atoms. Bond formation usually takes place in a strongly basic aqueous solution, but the process is also possible in acids, in which the reactive aluminosilicates are dissolved [8,9,10]. The most common alkaline activators are sodium hydroxide (NaOH) and sodium silicate (Na_2_SiO_3_, water glass) [11]. Experiments have shown that the quality of a geopolymer binder depends on several parameters, such as the activator concentration, the activator ratio, or the ratio of liquid to solid precursors [12,13,14,15]. According to the research papers, the optimal parameters for the production of the geopolymer are a liquid-to-solid ratio of 0.6 and an alkaline activator ratio of 2.5 [11].

Differences in the Na_2_SiO_3_ content affect the properties of geopolymers, such as hardening time, compressive strength, and workability [16]. The appropriate ratio of sodium hydroxide and sodium silicate in the alkaline activator improves the resistance of geopolymer materials to the harmful effects of sulfates [17]. Bocullo et al. examined the properties of the geopolymer depending on different SiO_2_/Na_2_O ratios (range 0.8–3.1). As an alkaline activator ratio of 2.5 results, the best compressive strength results were obtained for materials with a SiO_2_/Na_2_O ratio of 2.0 [18]. On the other hand, Wand et al.’s research determined the relationship between the Si/Al ratio and the properties of geopolymers—a geopolymer with a Si/Al ratio > 3 was characterized by worse stability in the air than the geopolymer with the Si/Al ratio < 2.5 [19].

Geopolymers are typically produced from aluminosilicate-rich raw materials [20]. Currently, the most commonly used raw materials in geopolymers are fly ash [21,22,23], blast furnace slags [24,25,26], or metakaolin [27,28]. Fly ash and slag are finished wastes from energy processes for use in geopolymerization. The advantage of these raw materials is that they do not require additional pretreatment before their alkaline activation. Unfortunately, these are raw materials that do not have fixed properties or chemical compositions. These properties depend on, among other things, the raw material used during the combustion process or the incineration technology used [4,29]. There is also the possibility of alkaline activation of other raw materials considered to be waste from mining, processing, and other industries, i.e., the energy industry. Examples of alkaline-activated raw materials that the researchers have used in their research include volcanic tuff [30,31], mine waste [32], or plasma waste [33,34,35]. Hypo slime (waste cellulose material from the paper industry) has proven to be a great alternative to replacing cement. In addition, it can be potentially used as a material for structural elements [36,37]. Research conducted on geopolymer materials has shown that they have excellent properties in terms of resistance to high temperatures [38], high compressive (from 25 MPa to more than 100 MPa) and flexural strength (from 5 MPa to 25 MPa) [39], high resistance to acids, sulfates, and chlorides [40], very high frost resistance [41], or absence or occurrence of slight shrinkage during setting [42]. In recent years, research has been conducted on the use of toxic and unfriendly waste as a precursor for the geopolymerization process. Lach et al. investigated the possibility of using municipal waste incineration plant waste as a precursor in a geopolymerization process to immobilize waste. The results showed a high level of immobilization of compounds and elements such as chlorides, sulfates, fluorides, barium, and zinc [43].

The motivation for the research work is to use waste streams from the mining and processing industry, as well as other industries such as the energy industry, as raw materials for the synthesis of modern geopolymer binders. This will not only represent a positive environmental aspect but will also contribute to the development of a circular economy. Furthermore, the use of post-production industrial waste will reduce the amount of waste in landfills and contribute to recycling. In this study, an attempt was made to use industrial waste to produce geopolymer binders. The work included structural and strength studies conducted to analyze and select potential precursors that could form the basis of alkali-activated binders.

The work aims to synthesize new geopolymer composites for use as construction binders using locally occurring waste material. The produced binders are based on waste materials and could potentially find application in conventional construction as replacement products for traditional Portland cement-based construction binders.

## 2. Materials and Methods

### 2.1. Materials and Samples Preparation

This paper presents the results of work related to the production of geopolymer binders based on waste materials such as chalcedonite (Ch), fly ash from lignite combustion (PB), diatomite dust calcined at 900 °C (D), and amphibolite (A). Metakaolin (M) was used as an additional modifier for binders based on waste materials. Chalcedonite (CRUSIL, Inowłódz, Poland) in the form of dust was obtained by milling to obtain a grain size of approximately 10 µm. Fly ash was obtained from the combustion of lignite at the Bełchatów Power Plant (Bełchatów, Poland). These ashes are characterized by a relatively high calcium content [24,25]. Another base material used for the production of alkali-activated binders is diatomite dust, obtained from the open-pit diatomite mine in Jawornik Ruski (Zohatin, Poland). Based on previous studies on diatomite material [26], it was decided to carry out a calcination process at 900 °C, and then the precursor thus prepared was used to synthesize binders. Amphibolite (Ogorzelec, Kamienna Góra, Poland) was subjected to a grinding process to obtain the appropriate grain gradation. The last material used to modify alkali-activated binder mixtures was metakaolin (Keramost, Kadaň, Czech Republic).

Geopolymer binders were prepared using a sodium activator and five types of base precursors, mixed in different weight ratios. The activation process was carried out with a 10-mol sodium hydroxide solution, NaOH, combined with a solution of sodium silicate (water glass, in a weight ratio of 1:1.5), which is the most commonly used hydroxide activator in geopolymer synthesis and is also the cheapest and most widely available of the alkali hydroxides. For the production of geopolymer masses, technical sodium hydroxide in the form of flakes (PCC Rokita SA, Brzeg Dolny, Poland) and an aqueous solution of sodium silicate R-145 (STANLAB, Gliwice, Poland) with a 2.5-mol modulus and a density of approximately 1.45 g/cm^3^ were used. Distilled water was used to prepare the sodium base. The alkaline solution was prepared by mixing an aqueous solution of sodium silicate with a 10 M sodium hydroxide solution. The solution was mixed and allowed to stabilize in temperature and equilibrate concentrations for 24 h. The prepared solution was then mixed with the precursors in a preset amount by weight (Table 1). After obtaining a homogeneous mass with a thick, plastic consistency, the mixtures were transferred to a set of 10 mm × 10 mm × 60 mm molds, which were then placed on a vibrating table to remove air bubbles. The molded geopolymer binders were cured in a laboratory dryer (SLW 750 STD, POL-EKO-APARATURA) for 24 h at 60 °C under atmospheric pressure. After 24 h, the samples were removed from the molds. Table 1 shows the composition of the prepared alkali-activated binders.

**Figure 1:**
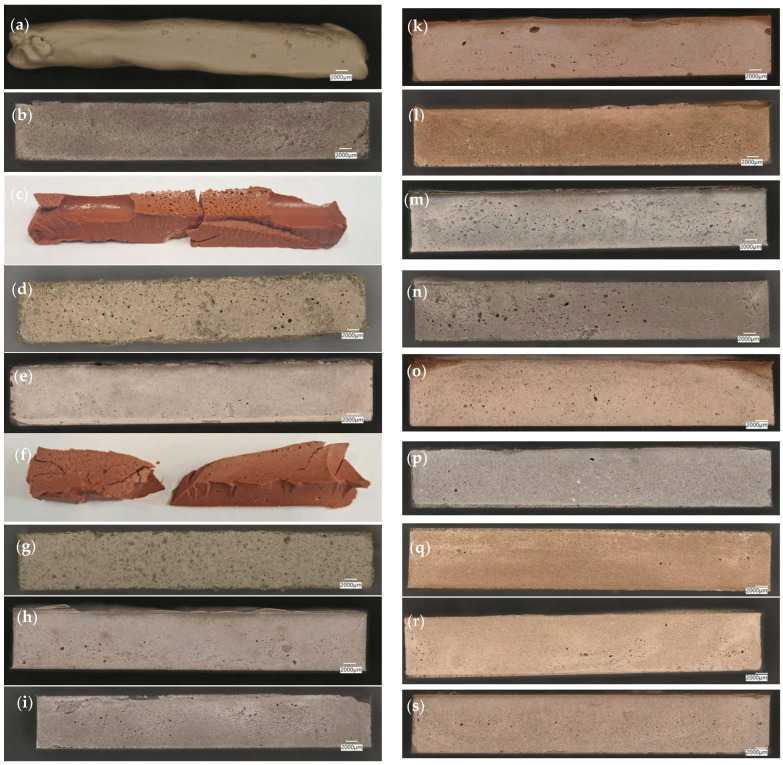

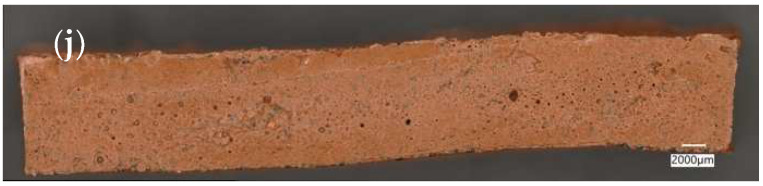
Obtained geopolymer binders based on waste materials: (**a**) R.Ch; (**b**), R.PB; (**c**) R.D; (**d**) R.A; (**e**) R.M; (**f**) Ch + D; (**g**) Ch + A; (**h**) Ch + M; (**i**) Ch + PB; (**j**) D + A; (**k**) D + M; (**l**) D + PB; (**m**) A + M; (**n**) A + PB; (**o**) Ch + D+A + M; (**p**) Ch + A + M + PB; (**q**) Ch + D + A + PB; (**r**) D + A + M + PB; (**s**) Ch + D + M + PB.

The metakaolin used in the mixtures was only a modifier of the individual waste-based binders. It can be seen that the content of the alkali solution varied depending on the type of mix. In some cases, a higher amount of alkali solution was necessary to achieve the right workability and consistency of the binder mixtures [23,44]. Figure 1 shows images of prepared alkali-activated materials.

In binders based on 100% chalcedonite (R.Ch), 100% amphibolite (R.A), a mixture of 50% amphibolite and 50% calcined diatomite (D + A) and a mixture of 50% chalcedonite and 50% amphibolite (Ch + A), despite using the same heating parameters as in the other binder mixtures, complete hardening of the material did not occur. Due to this fact, mechanical testing was not possible for this type of binder. Furthermore, in the case of a binder based on 100% calcined diatomite (R.D) and a mixture of 50% chalcedonite and 50% diatomite (Ch + D), despite the binders curing, the samples cracked during the demoulding process (Figure 1). As in the case of the R.Ch., R.A, D + A, and Ch + A materials, this resulted in the inability to carry out strength tests.

### 2.2. Research Methods

#### 2.2.1. Particle Size Analysis

Particle size analysis was carried out using an Anton-Paar PSA 1190LD laser particle size analyzer (AntonPaar GmbH, Graz, Austria). The measuring range of the apparatus is from 0.04 µm to 2500 µm. The test was conducted using the wet method with distilled water as the dispersing agent. Five measurements were carried out for each material, and then the average of the results was calculated using Kalliope Professional software (version 2.22.1, AntonPaar GmbH, Graz, Austria) and presented in the form of a plot of the average particle size distribution and the average cumulative curve. The precursors that formed the basis of the alkali-activated binders—chalcedonite, calcined diatomite, amphibolite, metakaolin, and fly ash from Bełchatów—were analyzed.

#### 2.2.2. Chemical and Mineralogical Composition of Precursors

The PANalytical Aeris instrument (Malvern PANalytical, Lelyweg 1, Almelo, Holan-dia) was used to investigate the phase composition of the base materials. Quantitative analysis was performed using the Rietveld method [29], which was implemented in the HighScore Plus software (version: 4.8, Malvern PANalytical B.V., Almelo, The Netherlands). The PDF-4+ database of the International Centre for Diffraction Data (ICDD) was used during the analysis. Measurements were recorded in the range 10–100° with a step of 0.003° (2θ) and a time per step of 340 s, using Cu Kα radiation.

The base materials were analyzed by fluorescence X-ray analysis (EDX) to determine the oxide composition. The analysis was performed on a SHIMADZU EDX-7200 (SHIMADZU Europa GmbH, Duisburg, Germany). The test was carried out in an air atmosphere with holders dedicated to bulk materials and with Mylar film.

#### 2.2.3. Strength Tests

Flexural strength tests were carried out according to EN 1015-11:2020-04 (Determination of flexural and compressive strength of hardened mortar) [45] on an MTS Criterion Model 43 universal testing machine (MTS System Corp., Eden Prairie, MN, USA) within a measuring range of up to 30 kN using an MTS axial extensometer. The speed was set at 10 mm/min. The flexural strength test specimens were made as 10 mm × 10 mm × 60 mm cuboids.

Compressive strength testing was carried out in accordance with the provisions of PN-EN 1015-11:2020-04 (Determination of flexural and compressive strength of hardened mortar) [45]. The test was carried out on an MTS Criterion Model 43 strength machine (MTS System Corp., Eden Prairie, MN, USA), with a measurement range of up to 30 kN. The speed was set at 10 mm/min. The specimens for compressive strength testing came from a bending test (half beams), which were placed between metal plates measuring 10 mm × 10 mm. A similar procedure for conducting compressive strength testing is also practiced by other researchers [46,47].

#### 2.2.4. Microstructure

The base materials and alkali-activated binders were subjected to microscopic observation to characterize the structure formed. The study was carried out using a JEOL IT200 scanning electron microscope (JEOL Ltd., Peabody, MA, USA). Samples after mechanical property tests were used for analysis. Before testing, the surface of the samples was coated with a conductive gold layer using a DII-29030SCTR Smart Coater vacuum sputtering machine (JEOL Ltd., Peabody, MA, USA).

## 3. Results and Discussion

### 3.1. Particle Size Analysis

**Figure 2:**
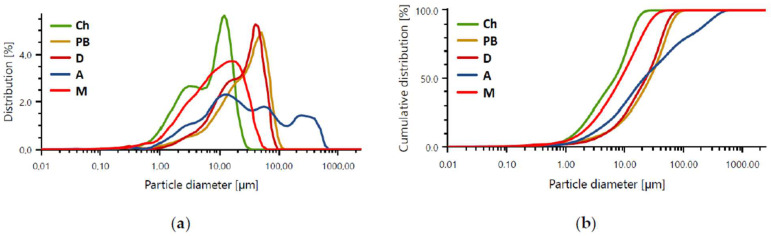
Average results of laser particle size analysis: (**a**) histogram of size distribution of individual precursors: Ch—chalcedonite, PB—fly ash from Belchatow, D—diatomite dust, A—amphibolite, M—metakaolin; (**b**) cumulative particle size distribution curves of the respective precursors.

Figure 2 and Table 2 show the particle size distribution histogram and cumulative particle size distribution curves as a function of particle percentage for the selected base materials. The results are presented as a plot of the mean particle size distribution and the mean cumulative curve (Figure 2).

The particle size distribution of the individual precursors has the character of a Gaussian diagram [48]—except for amphibolite analysis. This is most evident in the case of metakaolin and fly ash from Bełchatów. The curve characteristics for chalcedonite and calcined diatomite are similar.

**Figure 3:**
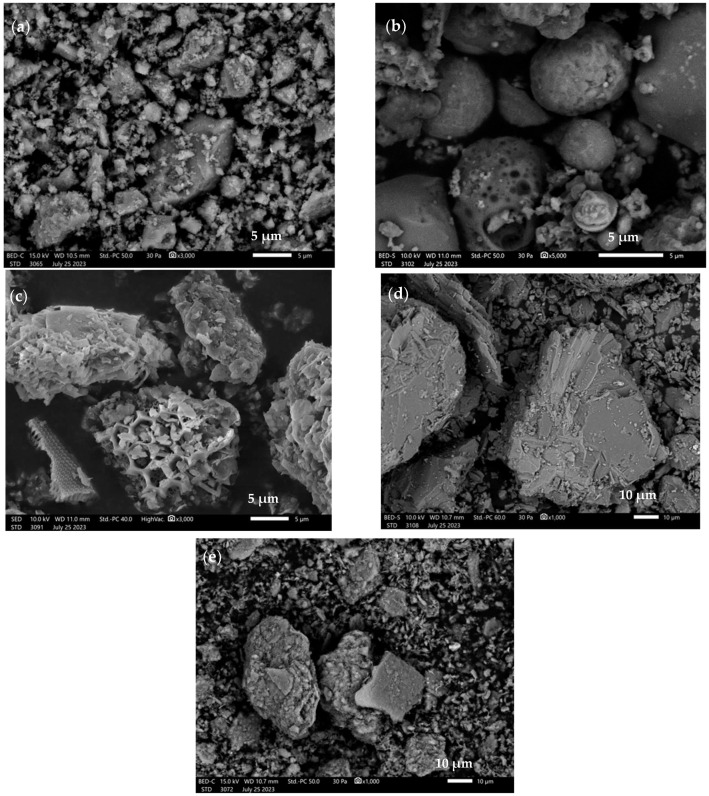
SEM images of base materials: (**a**) chalcedonite; (**b**) fly ash from Bełchatów; (**c**) calcined diatomite; (**d**) amphibolite; (**e**) metakaolin.

The average particle sizes of all base materials oscillate between 7 and 117 µm. The particle size analysis for chalcedonite (Table 2) showed that the average value of all the material’s particles oscillates around 7.99 µm. Particles with an average size of 1.52 µm represent 10% of the total volume of the sample tested. The particle size analysis for ash from Bełchatów (Table 2) showed that the average value of all ash particles oscillates within 32.43 μm. Particles with an average size of approximately 4.63 μm constitute 10% of the total volume of the sample tested. Particle size analysis for diatomite calcined at 900 °C (Table 2) showed that the average value of all particles oscillates around 27.24 μm. Particles with an average size of 4.49 μm represent 10% of the total volume of the sample tested. The particle size analysis for ground amphibolite (Table 2) shows that the mean value of all particles oscillates within 27.24 μm. Particles with an average size of 3.09 μm make up 10% of the total volume of the test sample. The analysis of the particle size of metakaolin (Table 2) showed that the mean value of all particles oscillates around 11.41 μm. Particles with an average size of 1.86 μm make up 10% of the total volume of the test sample. Figure 3 shows the morphology of the selected base materials.

Analysis of the base materials by scanning electron microscopy confirms the average results of laser particle size analysis. The microphotographs show that the chalcedonite (Figure 3a) particles oscillate around an average size of 8 µm. They are quite irregular, with sharp edges. Figure 3b shows fly ash particles from Bełchatów. Fly ash particles are spherical, with an average size of around 30 um. In addition, some particles are characterized by a porous structure. Compared to silica ash, limestone fly ash grains are characterized by very large particles of unburned carbon, which are porous and poorly sintered [49]. Figure 3c shows diatomite particles calcined at 900 °C. The average particle size oscillates around 50 µm. Similar structures have been analyzed by other researchers in their work [50,51]. Figure 3d shows amphibolite particles with diameters of over 100 µm. These particles are characterized by an irregular surface and sharp edges. This may be due to the grinding process of the amphibolite grains in the rotary mill. Figure 3e shows metakaolin particles that oscillate around 20 µm in diameter. The particles are irregular in surface and have sharp edges.

### 3.2. Chemical and Phase Compositions

The chemical composition of the tested precursors was determined by XRF. The results are shown in Table 3. All the alkali-activated binder base materials tested were mainly contained in their composition: SiO_2_, Al2O_3_, Fe_2_O_3_, SO_3_, K_2_O, TiO_2,_ or CaO.

Chalcedonite, in its composition, contains more than 98% SiO_2_, just under 1% Al_2_O_3,_ and SO_3_ at less than 0.5%. Similar material has also been studied by other researchers, who have shown similar chemical composition results in their work [52,53]. Fly ash from the combustion of lignite at the Bełchatów heat and power plant is classified as calcium ash. Calcium ash in the glassy phase is characterized by a high content of silica and aluminum. [54]. Examination of the chemical composition confirmed this assumption—the CaO content of the fly ash tested was over 32% and Al_2_O_3_ over 26%. In addition, the fly ash from Bełchatów also contained SiO_2_ in its composition, with a content of over 16%, Fe_2_O_3_ with over 12%, SO_3_ with over 10%, and TiO_2_ with about 0.5%. Diatomite calcined at 900 °C in its composition contained over 79% SiO_2_, over 12% Al_2_O_3_, over 4% Fe_2_O_3_, and over 2% K_2_O. Marczyk et al. studied calcined diatomite in their work. They obtained similar results in chemical composition [50]. In the amphibolite, which was subjected to chemical composition studies, the highest values were obtained for such compounds as SiO_2_—over 60%, Fe_2_O_3_—over 15%, Al_2_O_3_—over 13%, and CaO—over 7%. Maliszewski et al. analyzed amphibolite fossils from various deposits, including Ogorzelec. The results indicated that the highest values were represented by such compounds as SiO_2_, Fe_2_O_3_, Al_2_O_3,_ and CaO [55]. The chemical composition of metakaolin indicated that it has its own composition: SiO_2_—over 54%, Al_2_O_3_—over 41%, K_2_O—over 1%, and Fe_2_O_3_—over 1%. The same metakaolin has also been studied by other researchers, who obtained similar chemical composition results for metakaolin [56].

The phase composition of the tested precursors was determined by XRD. The results are shown in Table 4.

The X-ray diffraction method for chalcedonite showed that it contains 100% silicon oxide (SiO_2_) in its phase composition. In this case, no other phases were identified. Chalcedonite, studied by Vyšvařil in its phase composition, showed almost 99% SiO_2_ and about 1% Al_2_O_3_ [53]_._ Differences in the analysis may be due to the study of chalcedonite from different deposits (Poland, Czech Republic) or the reference sample analyzed. Phase composition analysis for fly ash from Belchatow showed the presence of such phases as Gehlenite (Ca_2_Al_2_SiO_7_)—more than 34%, Anhydrite (Ca(SO_4_)—more than 19%, Albite (NaAlSi_3_O_8_)—more than 17%, Hematite (Fe_2_O_3_)—almost 19%, Silicon Oxide (SiO_2_)—almost more than 5%, and Lime (CaO)—almost 5%. Fluid fly ash from Belchatow contains a great deal of calcium compounds in its composition [57], which was confirmed by phase analysis. The X-ray diffraction method for calcined diatomite at 900 °C showed that the phase composition was as follows: Silicon Oxide (SiO_2_)—40%, Kaolinite (Al_2_(Si_2_O_5_(OH)_4_))—more than 26%, Albite (NaAlSi_3_O_8_)—more than 20%, Illite-2R ((K, H_3_O)Al_2_Si_3_AlO_10_(OH)_2_)—more than 12%, and Hematite (Fe_2_O_3_)—1%. Phase analysis by Zheng et al. for calcined diatomite at 900 °C also showed the highest phase percentage for SiO_2_ [58]. Amphibolite is a metamorphic rock that mainly contains hornblende and plagioclase in its mineral composition, with variable amounts of anthophyllite, quartz, garnet, mica, and epidote [59]. Mineralogical analysis of the amphibolite sample showed the presence of such phases as anthophyllite (Mg_7_(Si_8_O_22_(OH_2_)))—over 43%, silicon oxide (SiO_2_)—over 26%, pargasite (NaCa_2_Mg_4_A_l3_Si_6_O_22_(OH)_2_)—over 18%, and magnesio-ferri-hornblende (formula in Table 4)—over 12%. The last precursor tested was metakaolin. In its phase composition, it contained, respectively: Kaolinite (A_l2_(Si_2_O_5_(OH)_4_))—more than 45%, Silicon Oxide (SiO_2_)—more than 26%, Illite-2R ((K, H_3_O)Al_2_Si_3_AlO_10_(OH)_2_)—more than 13%, and Muscovite (KAl_2_(Si_3_Al)O_10_(OH, F)_2_)—more than 14%. In their work, Morsy et al. analyzed metakaolin, which contained Kaolinite (highest content), Quarz, Illite, and Hematite [60].

### 3.3. Mechanical Properties and Structure Observation

Mechanical investigations, i.e., compressive or flexural strength, are one of the basic criteria for assessing the correctness of the geopolymerization process and for evaluating the potential suitability of the synthesized binder for construction applications [61]. The compressive strength of alkali-activated materials depends on several different variables, such as the structure of the material or the presence of a crystalline phase. In addition, the distribution and strength of the insoluble Al-Si particles and the reaction occurring at the surface between the gel phase and the insoluble Al-Si particles also influence the strength values of the geopolymer mortar [4,62]. The strength values are also influenced by the additives used and the base raw materials for geopolymers [63].

**Figure 4:**
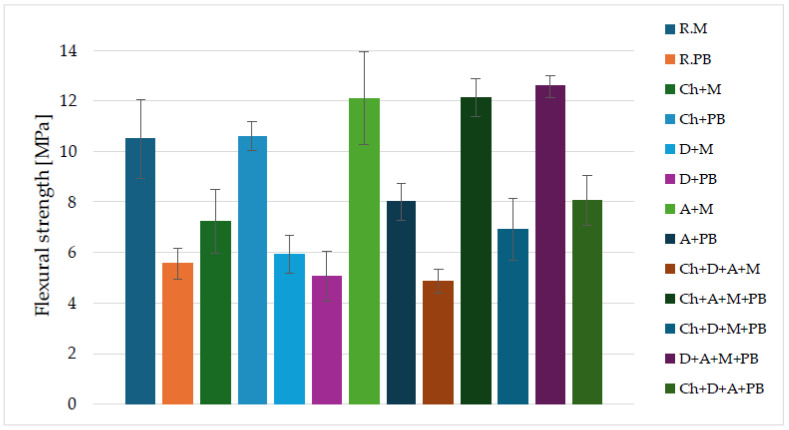
Flexural strengths for different binders based on alkali-activated waste materials.

Figure 4 shows the average flexural strength results for the alkali-activated binders produced.

The highest flexural strength values were achieved by a binder based on calcined diatomite, amphibolite, metakaolin, and fly ash from Bełchatów (D + A + M + PB)—12.58 MPa. Slightly lower flexural strength values were obtained for binders based on chalcedonite, amphibolite, metakaolin, and fly ash from Bełchatów (Ch + A + M + PB) and those based on amphibolite and metakaolin (A + M)—12.14 MPa and 12.11 MPa, respectively. The lowest compressive strength values were obtained for binders based on chalcedonite, calcined diatomite, amphibolite, and metakaolin (Ch + D+A + M) and calcined diatomite and fly ash from Belchatow (D + PB)—4.86 MPa and 5.06 MPa, respectively. Other flexural strength results for individual types of alkaline-activated binders were as follows: R.M—10.51 MPa, R.PB.—5.56 MPa, Ch + M—7.23 MPa, Ch + PB—10.62 MPa, D + M—5.92 MPa, A + PB—8.01 MPa, Ch + D + M + PB—6.93 MPa, and Ch + D + A + PB—8.07 MPa.

**Figure 5:**
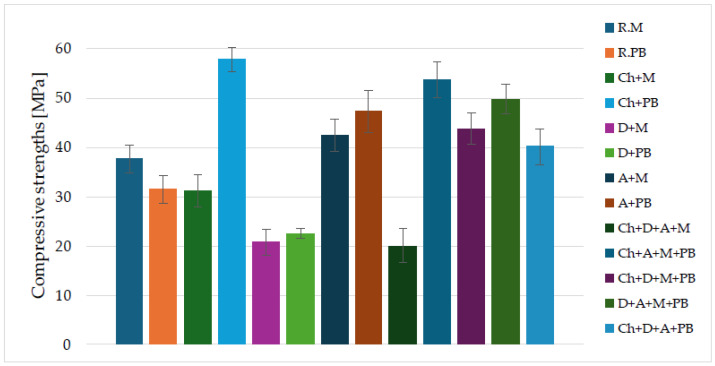
Compressive strengths for different binders based on alkali-activated waste materials.

Figure 5 shows the average compressive strength results for the alkali-activated material mixtures. Researchers’ results have shown that high-calcium fly ash is suitable as a base material for geopolymer binders [28,64].

Most of the alkali-activated binders achieved average compressive strengths of over 30 MPa. The highest compressive strength results were obtained with a binder based on chalcedonite and fly ash from Belchatow (Ch + PB)—57.74 MPa. Slightly lower compressive strength values were achieved by a binder based on chalcedonite, amphibolite, metakaolin, and fly ash from Bełchatów (Ch + A + M + PB)—53.61 MPa. The lowest values of compressive strength were obtained by a binder based on chalcedonite, calcined diatomite, amphibolite, and metakaolin—20.02 MPa and a binder based on calcined diatomite and metakaolin—20.70 MPa. Other compressive strength results for individual types of alkaline-activated binders were as follows: R.M—37.59, MPa, R.PB.—31.39 MPa, Ch + M—31.17 MPa, D + PB—22.50 MPa, A + M—42.42 MPa, A + PB—47.14 MPa, Ch + D + M + PB—43.77 MPa, D + A + M + PB—49.70 MPa, Ch + D + A + PB—40.04 MPa. To better present the results of the mechanical tests, they have been summarized in the form of a table (Table 5).

Fiertak and Stryszewska’s work investigated the compressive strength of Portland cement-based binders. The compressive strength of a sample based on cement alone was 30.3 MPa after 28 days. The 10% addition of silica fume increased the compressive strength to 35.5 MPa [65]. Özsoy et al. investigated the effect of diatomite dust on the strength properties of fly ash-based geopolymer mortars. The addition of 2% diatomite dust increased the compressive strength and flexural strength [66]. In another study, researchers investigated the effect of using chalcedonite powder as a partial substitute for cement in mortars. The best strength results were obtained when the cement was replaced with chalcedonite dust in amounts of 5% and 20% [67]. Cruz et al. investigated the effect of the addition of recycled gypsum in tricomponent binders (lime + metakaolin + gypsum). The addition of recycled gypsum increased the compressive strength and flexural strength of three-component binders [68].

Scanning electron microscopy (SEM) provides detailed topographic data and allows the evaluation of structures that cannot be revealed by other methods [69].

**Figure 6:**
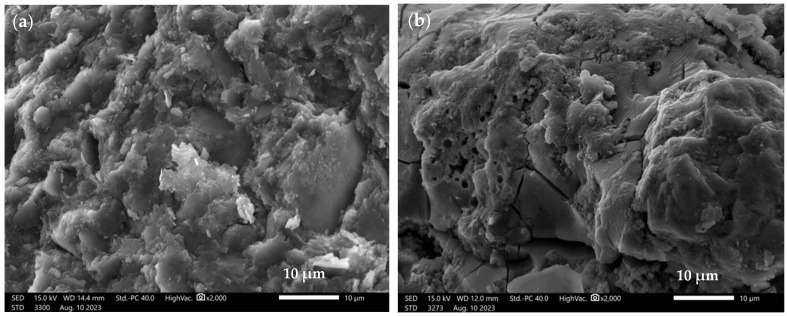
SEM images of alkali-activated binders based on 100%: (**a**) metakaolin—R.M; (**b**) fly ash from Bełchatów—R.PB, at 2000× magnification.

Figure 6 shows the microstructure of alkali-activated binders based on 100% waste materials—Figure 6a—binder based on metakaolin (R.M), Figure 6b—binder based on fly ash from Bełchatów (R.PB) at 2000× magnification.

The microstructure of the 100% metakaolin-based binder (Figure 6a) is characterized by a compact and amorphous structure. There are also no discernible pores in the formed material, but individual particles of undissolved metakaolin can be seen, with dimensions of approximately 20–30 µm. A similar microstructure of a metakaolin-based geopolymer was obtained in their work by Yang et al. [70]. Figure 6b shows the microstructure of the binder based on 100% calcium fly ash from Bełchatów (R.PB). Similar to the R.M. material, the microstructure of the material is compact. However, individual pores with a diameter of approximately 2–3 µm can be observed. The numerous cracks that occur in the topography of the material are the result of the strength tests carried out. In addition, unreacted fly ash particles from Bełchatów with a diameter of approximately 30–40 µm are present in the R.PB material, which is characterized by a porous structure. In comparison to silica ash, limestone fly ash grains are characterized by very large unburned carbon particles and a porous and poorly sintered structure [71]. Bąk et al. studied geopolymers based on fly ash from Bełchatów and sand. They obtained a geopolymer structure, which was also characterized by undissolved ash particles and a compact and amorphous structure [72].

Microstructure studies provide complementary knowledge about the mechanical properties of the tested materials [73,74,75].

**Figure 7:**
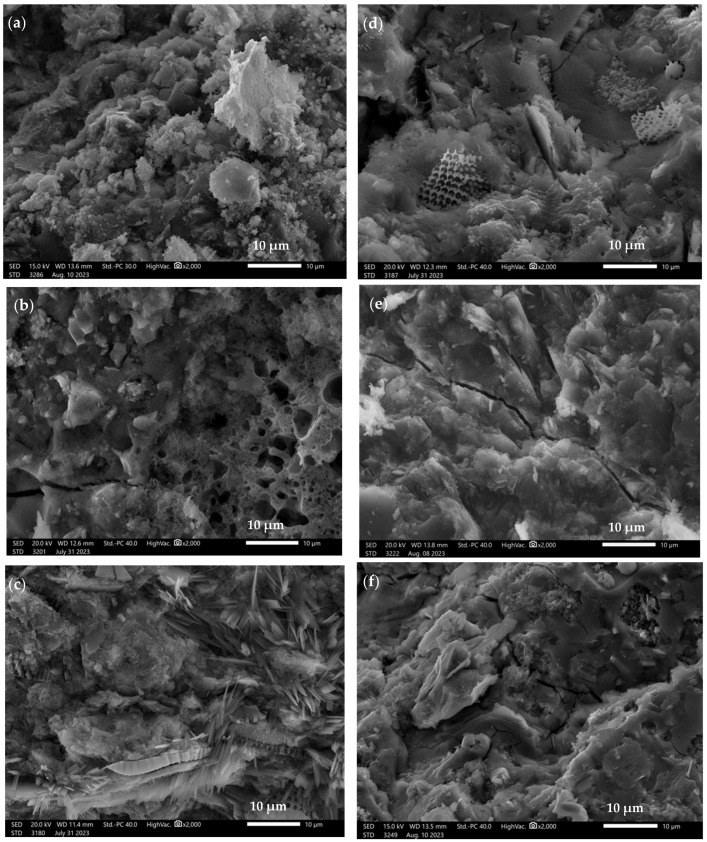
SEM images of alkali-activated binders based on 50–50% mixtures: (**a**) chalcedonite + metakaolin—Ch + M; (**b**) chalcedonite + fly ash from Belchatów—Ch + PB; (**c**) calcined diatomite + metakaolin—D + M; (**d**) calcined diatomite + fly ash from Belchatów—D + PB; (**e**) amphibolite + metakaolin—A + M; (**f**) amphibolite + fly ash from Belchatów—A + PB, magnified by 2000×.

Figure 7 shows the microstructure of alkali-activated binder blends based on various waste materials—fly ash from Belchatów, chalcedonite, diatomite calcined at 900 °C, amphibolite, and metakaolin. The activated materials were mixed in a 50–50% weight proportion in different variations.

Figure 7a shows the microstructure of a binder based on a mixture of chalcedonite and metakaolin—Ch + M. The microstructure of the binder is compact, with a few undissolved chalcedonite particles of about 7 µm in size. No distinct pores are seen in the investigated microstructure. Figure 7b shows the microstructure of a binder based on chalcedonite and fly ash from Bełchatów—Ch + PB. The microstructure of the material is compact and amorphous. However, individual pores with a diameter of approximately 3–4 µm can be observed. The noticeable cracks present in the topography of the material are the result of the strength tests carried out. In addition, unreacted fly ash particles from Bełchatów with a diameter of approximately 40–60 µm are present in the Ch + PB material, which is characterized by a porous structure. Figure 7c shows the microstructure of a binder based on calcine diatomite and metakaolin. In this case, the surface morphology is quite irregular, with porous structures appearing on the surface (diatomite particles) with a size of about 10 µm. In addition, we can also see numerous needle-like structures of about 10µm in length. Figure 7d shows the microstructure of the binder based on calcite diatomite and fly ash from Bełchatów. The microstructure of the material is compact and amorphous, with numerous undissolved diatomite particles present, embedded in the binder matrix, whose size oscillates in the 10–15 µm range. In addition, we can detect cracks in the material, which are the result of strength testing, and a small number of pores with a diameter of approximately 1 µm. Figure 7e shows the microstructure of the binder based on a mixture of amphibolite and metakaolin—A + M. The microstructure of the binder is compact and amorphous. No clear pores can be seen in the investigated microstructure. The noticeable cracks present in the topography of the material are the result of the strength tests carried out. Figure 7f shows the microstructure of the binder based on amphibolite and fly ash from Bełchatów. The structure of the material is rather compact, with numerous undissolved particles of fly ash from Bełchatów, with a size oscillating around 10 µm. Pores with a diameter of 1–3 µm can also be seen in the microstructure of the binder. The material also shows numerous cracks, following mechanical testing.

**Figure 8:**
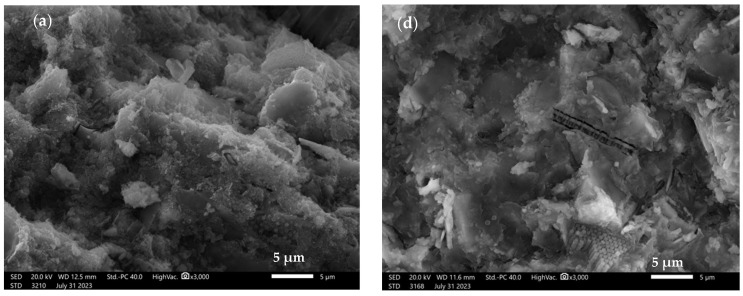

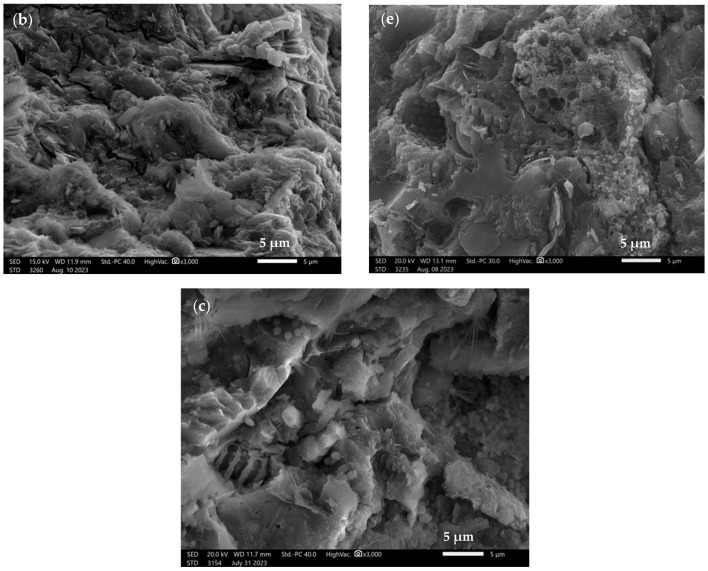
SEM images of alkali-activated based on 25%–25%–25%–25% mixtures: (**a**) chalcedonite + calcined diatomite + amphibolite + metakaolin—Ch + D+A + M; (**b**) chalcedonite + amphibolite + metakaolin + fly ash from Bełchatów—Ch + A + M + PB; (**c**) chalcedonite + calcined diatomite + amphibolite + fly ash from Bełchatów—Ch + D + A + PB; (**d**) calcined diatomite + amphibolite + metakaolin + fly ash from Bełchatów—D + A + M + PB; (**e**) chalcedonite + calcined diatomite + metakaolin + fly ash from Bełchatów- Ch + D + M + PB, magnification 3000×.

Figure 8 shows the microstructure of alkali-activated binders based on mixtures of waste raw materials, with the weight proportions of the various precursors in the ratio: 25%–25%–25%–25%.

Figure 8a shows the microstructure of a binder based on chalcedonite, calcined diatomite, amphibolite, and metakaolin (Ch + D + A + M). The microstructure investigated is compact and amorphous, with no obvious pores in its structure. Figure 8b shows the microstructure of a binder based on chalcedonite, amphibolite, metakaolin, and fly ash from Belchatów (Ch + A + M + PB). The microstructure studied is compact, with a lack of distinct pores in its structure. Particles of undissolved fly ash oscillating around 5–7 µm in size can be observed. Figure 8c shows the microstructure of a binder based on chalcedonite, calcined diatomite, amphibolite, and fly ash from Bełchatów (Ch + D + A + PB). The microstructure studied is compact, with a lack of distinct pores in its structure. Particles of undissolved calcined diatomite with a size oscillating around 5 µm can be observed. Figure 8d shows the microstructure of a binder based on calcined diatomite, amphibolite, metakaolin, and fly ash from Bełchatów (D + A + M + PB). The microstructure studied is compact, in which several distinct pores in the range of 1–2 µm can be observed. In the binder matrix, particles of undissolved calcined diatomite with a size oscillating in the range of 10–15 µm can be observed. Figure 8e shows the microstructure of a binder based on chalcedonite, calcined diatomite, metakaolin, and fly ash from Bełchatów (Ch + D + M + PB). The investigated microstructure is compact, in which several distinct pores oscillating around 5 µm can be observed. In addition, particles of undissolved fly ash from Belchatów are present in the microstructure—structures around 20–30 µm in size, with porous characteristics. Alehyen et al. investigated and described the microstructure of fly ash-based mortars. They described it as a heterogeneous, porous mixture in which not all fly ash particles were dissolved or partially dissolved [76]. The microstructure of geopolymers with the addition of metakaolin was studied in another paper by Yang et al. They found that after the addition of metakaolin, the content and pore diameter of the geopolymer decreased, which could be the reason for the improved mechanical properties [70]. Vyšvařil et al. on the partial replacement of lime binder with chalcedonite powder as a 0% to 40% lime replacement additive. With increasing chalcedonite content in the lime mortars, a decrease in the total porosity of the samples was observed, associated with a decrease in water absorption [53].

## 4. Conclusions

The aim of the above research was to explore the possibility of using waste raw materials found locally for use as alkali-activated binders. Binders based on such raw materials as chalcedonite, diatomite calcined at 900 °C, calcium fly ash from Bełchatów, amphibolite, and metakaolin were synthesized. This work included structural and strength studies conducted to analyze and select potential precursors that could form the basis of advanced alkali-activated binders. The results of the study show that:

1. The highest compressive strength values were achieved by a binder based on chalcedonite and fly ash from Bełchatów (Ch + PB)—567.74 MPa. Slightly lower compressive strength values were achieved by a binder based on chalcedonite, amphibolite, metakaolin, and fly ash from Belchatów (Ch + A + M + PB)—53.61 MPa.
2. The lowest values of compressive strength were obtained by a binder based on chalcedonite, calcined diatomite, amphibolite, and metakaolin (Ch + D+A + M)—20.02 MPa and a binder based on calcined diatomite and metakaolin (D + M)—20.70 MPa.
3. The highest flexural strength values were achieved by a binder based on calcined diatomite, amphibolite, metakaolin, and fly ash from Bełchatów (D + A + M + PB)—12.58 MPa.
4. Average flexural strength values were obtained at similarly high levels for binders based on chalcedonite, amphibolite, metakaolin, and fly ash from Belchatow (Ch + A + M + PB) and binders based on amphibolite and metakaolin (A + M)—12.14 MPa and 12.11 MPa, respectively.
5. The lowest flexural strength values were achieved by a binder based on chalcedonite, calcined diatomite, amphibolite, and metakaolin and (Ch + D+A + M)—4.86 MPa.
6. Raw materials such as chalcedonite, calcined diatomite, or amphibolite are not suitable for alkaline activation alone. However, the use of them in appropriate weight proportions as an addition to mixtures of other waste materials promotes the formation of a geopolymer binder with very good mechanical properties.

## Figures and Tables

**Table 1:** Composition of alkali-activated samples based on fly ash from Bełchatów (PB), chalcedonite (Ch), calcined diatomite dust (D), amphibolite (A), and metakaolin (M).

Index	Base Materials (S) [Weight Ratio]					Alkaline Activator (L)	Liquid/Solid [Weight Ratio]
	Ch	PB	D	A	M		
R.Ch	1	-	-	-	-	10 M NaOH + sodium water glass (weight ratio: 1: 1.5)	0.62/1
R.PB	-	1	-	-	-		1.22/1
R.D	-	-	1	-	-		1.01/1
R.A	-	-	-	1	-		0.31/1
R.M	-	-	-	-	1		0.75/1
Ch + D	1	-	1	-	-		0.82/1
Ch + A	1	-	-	1	-		0.34/1
Ch + M	1	-	-	-	1		0.61/1
Ch + PB	1	1	-	-	-		0.54/1
D + A	-	-	1	1	-		0.68/1
D + M	-	-	1	-	1		0.68/1
D + PB	-	1	1	-	-		0.82/1
A + M	-	-	-	1	1		0.48/1
A + PB	-	1	-	1	-		0.48/1
Ch + D+A + M	1	-	1	1	1		0.54/1
Ch + A + M + PB	1	1	-	1	1		0.61/1
Ch + D + A + PB	1	1	1	1	-		0.51/1
D + A + M + PB	-	1	1	1	1		0.48/1
Ch + D + M + PB	1	1	1	-	1		0.54/1

**Table 2:** Particle size average of base materials.

Index	D_10_	D_50_	D_90_	Mean Size [µm]	Standard Deviation [µm]
Ch	1.52	6.92	14.98	7.99	0.143
PB	4.63	26.76	63.12	32.43	1.05
D	4.49	22.26	52.56	27.24	0.03
A	3.09	33.84	327.49	116.12	1.87
M	1.86	8.36	23.68	11.41	0.39

**Table 3:** Oxide composition of analyzed base materials.

Ch		PB		D		A		M	
Compound Formula	Conc, %	Compound Formula	Conc, %	Compound Formula	Conc, %	Compound Formula	Conc, %	Compound Formula	Conc, %
SiO_2_	98.292	CaO	32.297	SiO_2_	79.297	SiO_2_	60.802	SiO_2_	54.504
Al_2_O_3_	0.923	Al_2_O_3_	26.251	Al_2_O_3_	12.033	Fe_2_O_3_	15.889	Al_2_O_3_	41.716
SO_3_	0.473	SiO_2_	16.761	Fe_2_O_3_	4.627	Al_2_O_3_	13.504	K_2_O	1.557
K_2_O	0.097	Fe_2_O_3_	12.843	K_2_O	2.491	CaO	7.155	Fe_2_O_3_	1.150
Fe_2_O_3_	0.095	SO_3_	10.781	TiO_2_	0.594	TiO_2_	1.234	TiO_2_	0.651
CaO	0.056	TiO_2_	0.569	CaO	0.441	SO_3_	0.688	CaO	0.168
TiO_2_	0.028	SrO	0.093	SO_3_	0.331	MnO	0.323	SO_3_	0.132
MnO	0.008	V_2_O_5_	0.088	MnO	0.064	K_2_O	0.243	V_2_O_5_	0.033
PtO_2_	0.007	MnO	0.087	V_2_O_5_	0.031	V_2_O_5_	0.064	MnO	0.016
Au_2_O	0.004	K_2_O	0.085	ZrO_2_	0.023	ZnO	0.026	ZrO_2_	0.016
HgO	0.004	ZnO	0.051	Cr_2_O_3_	0.015	SrO	0.020	Rb_2_O	0.011
V_2_O_5_	0.003	ZrO_2_	0.021	ZnO	0.012	Ag_2_O	0.014	SrO	0.009
CuO	0.002	Cr_2_O_3_	0.021	SrO	0.010	Cr_2_O_3_	0.014	Ga_2_O_3_	0.007
SeO_2_	0.002	Y_2_O_3_	0.016	CuO	0.009	CuO	0.010	Cr_2_O_3_	0.006
NiO	0.002	Ir_2_O_3_	0.013	Y_2_O_3_	0.006	Ir_2_O_3_	0.009	ZnO	0.005
ZrO_2_	0.002	CuO	0.011	Ir_2_O_3_	0.006	Y_2_O_3_	0.006	PbO	0.005
Y_2_O_3_	0.001	Bi_2_O_3_	0.009	NiO	0.004			CuO	0.003
SrO	0.001	SeO2	0.003	Ag_2_O	0.003			Y_2_O_3_	0.003
								Au_2_O	0.002
								NbO	0.002
								NiO	0.002
								GeO_2_	0.001

**Table 4:** Identified phases in base precursors.

Identified Phase	Chemical Formula	Amount of Phase [%]				
		PB	A	Ch	M	D
Gehlenite	Ca_2_Al_2_SiO_7_	34.1	-	-	-	-
Anhydrite	Ca(SO_4_)	19.3	-	-	-	-
Albite	NaAlSi_3_O_8_	17.3	-	-	-	20.1
Hematite, syn	Fe_2_O_3_	18.9	-	-	-	1.0
Silicon Oxide	SiO_2_	5.5	26.3	100	26.3	40.0
Lime, syn	CaO	4.9	-	-	-	-
Magnesio-ferri-hornblende	(Na_0.28_K_0.13_)(Ca_1.81_Mn_0.02_Fe_0.17_) (Mg_3.14_Fe_1.46_Ti_0.15_Al_0.25_)(Si_6.92_Al_1.08_O_22_)(OH)_2_	-	12.5	-	-	-
Anthophyllite	Mg_7_(Si_8_O_22_(OH_2_))	-	42.8	-	-	-
Pargasite, syn	NaCa_2_Mg_4_Al_3_Si_6_O_22_(OH)_2_	-	18.5	-	-	-
Kaolinite	Al_2_(Si_2_ O_5_(OH)_4_)	-	-	-	45.6	26.8
Illite-2R (NR)	(K,H_3_O)Al_2_Si_3_AlO_10_(OH)_2_	-	-	-	13.3	12.1
Muscovite	KAl_2_(Si_3_Al)O_10_(OH,F)_2_	-	-	-	14.8	-

**Table 5:** Summary of average compressive and flexural strength test results of geopolymer binders.

Sample	Compressive Strengths [MPa]	Flexural Strengths [MPa]
R.M	37.59	10.51
R.PB	31.39	5.56
Ch + M	31.17	7.23
Ch + PB	57.74	10.62
D + M	20.70	5.92
D + PB	22.50	5.06
A + M	42.42	12.11
A + PB	47.14	8.01
Ch + D+A + M	20.02	4.86
Ch + A + M + PB	53.61	12.14
Ch + D + M + PB	43.77	6.93
D + A + M + PB	49.70	12.58
Ch + D + A + PB	40.04	8.07

## Data Availability

Data are contained within the article.
